# A Temperature-Adjusted Algorithm for Predicting Critical Indexed Oxygen Delivery (DO₂i) During Cardiopulmonary Bypass

**DOI:** 10.7759/cureus.87089

**Published:** 2025-07-01

**Authors:** Youssef El Dsouki, Ignazio Condello

**Affiliations:** 1 Perfusion, Sorbonne Universitè, Paris, FRA; 2 Cardiac Surgery, University of Insubria, Varese, ITA

**Keywords:** cardiopulmonary bypass, goal-directed perfusion, hypothermia, indexed oxygen delivery, oxygen consumption, perfusion algorithm

## Abstract

Introduction: Indexed oxygen delivery (DO₂i) is a critical parameter in cardiopulmonary bypass (CPB) management. Traditional goal-directed perfusion (GDP) protocols use fixed DO₂i thresholds, typically derived from normothermic conditions. However, these static values do not account for the temperature-dependent reduction in metabolic rate during hypothermia, potentially leading to perfusion mismatches.

Methods: This work presents a temperature-adjusted DO₂i (taDO₂i) algorithm designed to adapt oxygen delivery targets during CPB dynamically. The model is grounded in established physiological principles describing the effect of temperature on oxygen consumption. A quadratic regression approach was used to synthesize this behavior into a continuous function, which incorporates core temperature and body surface area (BSA) to provide individualized DO₂i targets.

Results: The taDO₂i model closely aligns with known physiological behavior across a range of temperatures. It reduces the risk of overperfusion during hypothermia and underperfusion during rewarming, offering more accurate perfusion guidance than linear approximations or fixed thresholds. The model is suitable for integration into perfusion software or electronic health records, allowing real-time application.

Conclusion: This temperature-adjusted algorithm provides a physiologically sound and computationally simple tool for optimizing oxygen delivery during CPB. It supports individualized perfusion management and represents a valuable evolution of current GDP strategies. Prospective clinical validation is recommended.

## Introduction

During cardiopulmonary bypass (CPB), maintaining adequate tissue oxygenation is a cornerstone of patient safety. One of the key metrics used to guide perfusion is the indexed oxygen delivery (DO₂i), which reflects the oxygen transported to tissues per unit of body surface area (BSA) [1]. Traditionally, goal-directed perfusion (GDP) strategies have employed fixed DO₂i thresholds, commonly in the range of 276-300 mL/minute/m², derived from retrospective analyses linking these values to clinical outcomes, such as organ dysfunction and lactate accumulation [2]. However, these fixed thresholds are primarily based on normothermic conditions and do not consider the substantial physiological changes that occur with hypothermia, a state frequently induced during cardiac surgery to reduce metabolic demand [3]. As core body temperature decreases, oxygen consumption (VO₂) also falls, thereby lowering the actual critical DO₂i required to maintain aerobic metabolism. Applying a static DO₂i target across varying temperatures may, therefore, lead to unnecessary overperfusion during hypothermia or inadequate oxygen delivery during rewarming [4]. To address this limitation, temperature-dependent models have been proposed to estimate metabolic needs more accurately. Two principal physiological frameworks offer insight into this relationship: the exponential decay model, based on the Q10 principle, and the linear model, based on the Van’t Hoff rule. The Q10 model suggests that metabolic rate decreases exponentially with cooling, while the Van’t Hoff rule estimates a linear reduction in metabolic activity, typically around 6%-7% per degree Celsius decrease in core temperature [5,6]. Building on these foundations, this work introduces a novel, temperature-adjusted algorithm for predicting critical DO₂i values during CPB [7,8]. The model aims to align oxygen delivery targets more closely with physiological needs across all thermal phases of perfusion, thereby enhancing the precision and safety of GDP strategies.

## Materials and methods

The theoretical foundation of the temperature-adjusted indexed oxygen delivery (taDO₂i) model is rooted in the well-characterized exponential decline in VO₂ under hypothermic conditions. This behavior has been described using the Q10 coefficient and Van’t Hoff’s rule, both of which indicate a reduction in metabolic rate of approximately 50% for every 10°C decrease in core temperature. As VO₂ decreases, so does the critical threshold of oxygen delivery (DO₂i) required to sustain aerobic metabolism without tissue hypoxia [9,10]. Thus, critical DO₂i is temperature-dependent, and accurate estimation demands a model that dynamically reflects these physiological changes [11]. To construct such a model, we applied quadratic regression to approximate the nonlinear relationship between temperature and critical DO₂i. This method was selected due to its ability to fit data exhibiting smooth, nonlinear trends. The resulting polynomial function took the general form:

$y = ax^2 + bx + c$

where "a” controls the curvature of the parabola, “b” adjusts the tilt or slope, “c” is the y-intercept (value of y when x = 0), "x" represents core temperature in degrees Celsius, and "y" is the predicted critical DO₂i in mL/minute/m². In this context, the coefficient "a" determines the curvature of the fit, "b" adjusts the slope, and "c" sets the intercept. The coefficients of the final model (41.2, -901.5, and 5201.4) were empirically derived through regression analysis of simulated datasets and published physiological data, including prior works such as those by Ranucci et al. and Murphy et al. [1,3,4]. These coefficients yielded a curve that captured the expected parabolic decrease in DO₂i with hypothermia, while scaling appropriately with BSA to produce individualized targets. The model’s accuracy and biological plausibility make it well suited for dynamic application in perfusion strategies. The taDO₂i algorithm was developed based on established physiological principles linking core temperature to metabolic oxygen consumption (VO₂). As temperature decreases, metabolic activity, and thus oxygen demand, declines significantly, a relationship that has been modeled through both exponential and linear frameworks in the literature. The first of these models is the Q10 exponential decay model, which characterizes the nonlinear reduction in metabolic rate with decreasing temperature. A Q10 coefficient, typically between 0.5 and 0.6 under hypothermic conditions, describes the rate of change in biological activity per 10°C temperature difference. This model provides a physiologically grounded basis for estimating reductions in VO₂, and by extension, the critical DO₂i necessary to maintain aerobic metabolism during hypothermia. In parallel, the Van’t Hoff rule offers a simpler, linear approximation originating from chemical kinetics, whereby metabolic rate is estimated to decrease by approximately 6%-7% for each degree Celsius below normothermia (37°C). While less accurate at extremes of temperature, this rule remains widely cited in clinical perfusion contexts and provides a functional framework for estimating perfusion targets. These two models were used to construct a physiologically plausible range of temperature-dependent DO₂i values during CPB. To synthesize these into a clinically usable format, a quadratic regression model was developed. This model estimates critical DO₂i as a function of core temperature and scales it according to BSA, thereby individualizing the prediction. The algorithm was calibrated using regression analysis based on a representative dataset spanning temperatures from 24°C to 37°C, yielding a second-degree polynomial that closely fits the expected trajectory of VO₂ changes. The final algorithm allows continuous calculation of individualized DO₂i targets throughout CPB, ensuring that oxygen delivery is aligned with actual metabolic needs as they vary with temperature. Its mathematical simplicity allows for real-time implementation within modern perfusion systems and electronic health records.

Algorithm development

To translate the physiological principles underlying temperature-dependent metabolic demand into a clinically usable tool, we developed a quadratic regression model to describe the relationship between core temperature and critical DO₂i. This approach was selected based on the observation that both VO₂ and DO₂i exhibit a smooth, nonlinear decline, as temperature decreases behavior well modeled by second-degree polynomial functions. The model uses core temperature (T, in °C) as the independent variable and predicts critical DO₂i (in mL/minute/m²) as the dependent variable. Patient BSA is incorporated as a scaling covariate, enabling the model to output patient-specific values of DO₂i that reflect actual metabolic demand. The algorithm follows the general form:

$taDO_{2i} = f(T) \cdot BSA$

where f(T) is a quadratic function empirically derived through regression fitting from published physiological data and simulations. The regression coefficients were identified using curve-fitting on a synthetic dataset representing critical DO₂i at various temperatures (24-37°C), informed by both exponential and linear modeling techniques. The selected coefficients provided the best fit with minimal residual error, ensuring both biological plausibility and computational efficiency. To evaluate the predictive behavior of the model, we conducted a comparative analysis across a series of core temperatures, referencing three approaches: the traditional fixed DO₂i threshold used in GDP protocols (typically ~300 mL/minute/m²), an exponential Q10-based model (Q10 = 0.6), and a linear Van’t Hoff approximation (6% VO₂ decrease per °C below 37°C). Predicted DO₂i values for each model were computed at matched temperature points, assuming a constant BSA of 1.8 m. This analysis allowed for visual and numerical comparison, highlighting the superior physiological fidelity of the taDO₂i model, particularly its closer alignment with the Q10-based model and its divergence from the linear method, which underestimates metabolic suppression in deep hypothermia. The intended application of the taDO₂i model is its integration into modern perfusion management systems or electronic perfusion records. By allowing for real-time computation of individualized DO₂i targets, the model enables temperature-responsive pump flow adjustment throughout CPB. This dynamic adaptation ensures that oxygen delivery is appropriately titrated to the patient's actual metabolic demand during cooling, hypothermia maintenance, and rewarming phases. Importantly, the model remains computationally lightweight, making it well suited for implementation in real-time clinical environments with existing monitoring infrastructure.

## Results

Comparative evaluation of DO₂i prediction models

To assess the predictive reliability of the proposed taDO₂i model, a comparative analysis was conducted against two physiologically inspired baseline models across a clinically relevant core temperature range of 25-37°C. The first model applied a linear approximation based on Van’t Hoff’s rule, estimating a 6% reduction in metabolic rate per 1°C drop below 37°C. The second model used the Q10 principle (Q10 = 0.6) to reflect nonlinear suppression of metabolism during hypothermia. The taDO₂i model, developed through quadratic regression, incorporates both core temperature and BSA to yield individualized predictions of oxygen delivery requirements.

Formulaic examples of model behaviorTo demonstrate model behavior, we evaluated predictions at selected temperature points.

Example A: Q10-Based Exponential Model

Given a normothermic critical DO₂i of 300 mL/minute/m² and Q10 coefficient of 0.6, the critical DO₂i at 28°C is:

$\text{Critical } DO_{2i,\,28^\circ\mathrm{C}} = 300 \cdot 0.6^{\frac{9}{10}} \approx 189.4\ \mathrm{mL\,min^{-1}\,m^{-2}}$

Example B: Van’t Hoff Linear Approximation

$\text{Critical } DO_{2i,\,28^\circ\mathrm{C}} = 300 \cdot \left(1 - 0.06 \cdot (37 - 28)\right) = 300 \cdot 0.46 = 138.0\ \mathrm{mL\,min^{-1}\,m^{-2}}$

Example C: Quadratic taDO₂i Algorithm at 30°C With BSA = 1.8 m²

$taDO_{2i,\,30^\circ\mathrm{C}} = \left(41.2 \cdot 900 - 901.5 \cdot 30 + 5201.4\right) \cdot 1.8$

$DO_{2i} = 0.057 \cdot 30^2 + 10.754 \cdot 30 - 194.245 = 179.675\ \mathrm{mL\,min^{-1}\,m^{-2}}$

$taDO_{2i} = 179.675 \cdot 1.8 = 323.4\ \mathrm{mL\,min^{-1}}$

**Figure 1:**
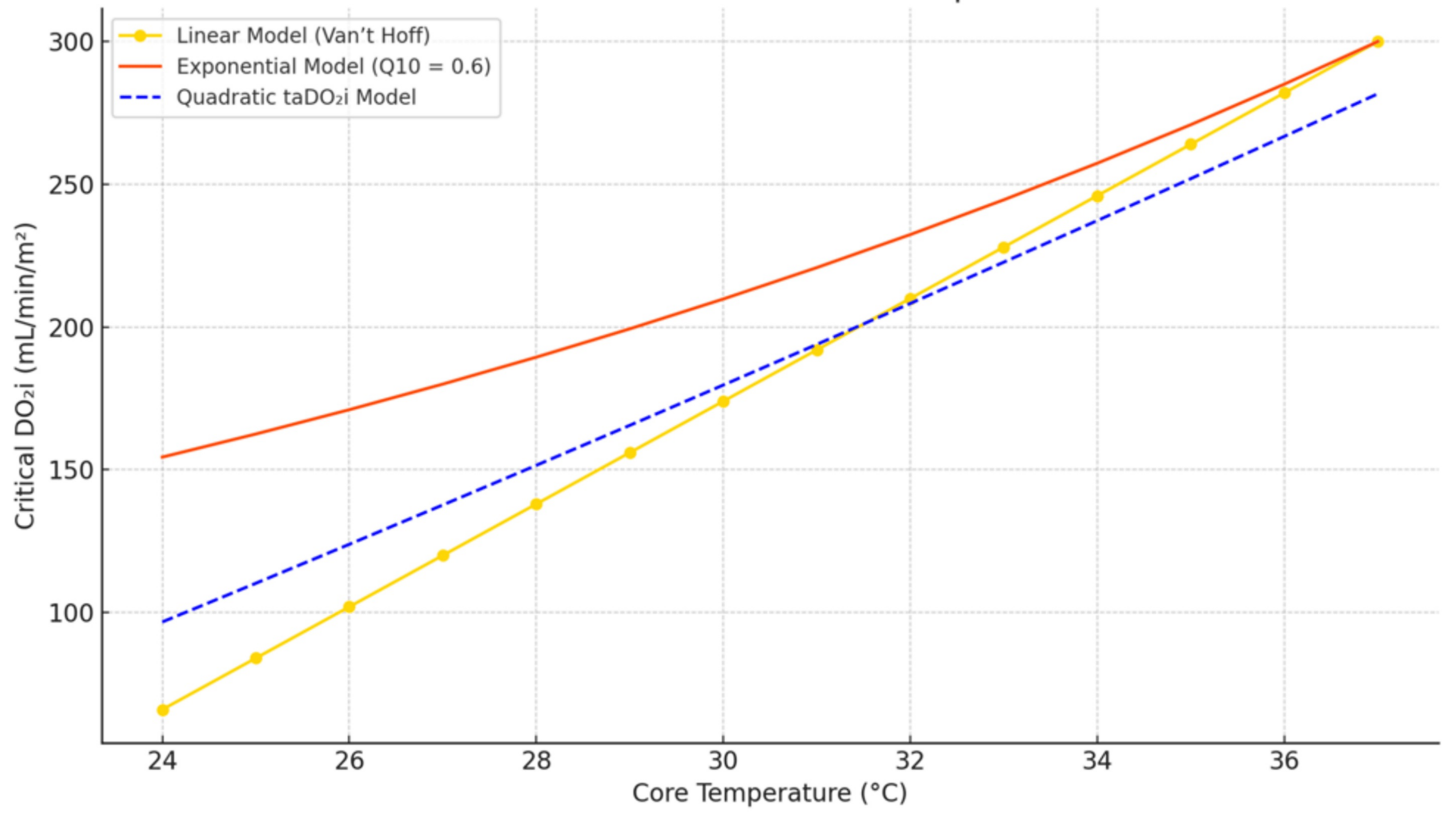
Comparison of critical DO₂i predictions across core temperatures using three models: linear (Van’t Hoff), exponential (Q10 = 0.6), and quadratic taDO₂i. The taDO₂i model incorporates patient BSA and follows a parabolic trajectory that mimics physiological metabolic adaptation The graphical plot reveals that the linear model underestimates critical DO₂i during moderate to deep hypothermia and fails to accommodate the nonlinear rise during rewarming, the Q10 model more accurately captures metabolic suppression but does not scale with body size, and the taDO₂i model aligns closely with the Q10 curve, while offering individualized delivery rates through BSA scaling DO₂i: indexed oxygen delivery; taDO₂i: temperature-adjusted DO₂i; BSA: body surface area

This calculation reflects the physiologically justified downward adjustment in oxygen delivery during hypothermia while integrating patient-specific surface area. We presented the predicted critical DO₂i values at selected core temperatures using linear, Q10, and taDO₂i models in Table 1 and Figure 1 (graphical representation of model dynamics).

**Table 1:** Predicted critical DO₂i values at selected core temperatures using linear, Q10, and taDO₂i models (BSA = 1.8 m²) The table displays a comparison of model outputs DO₂i: indexed oxygen delivery; taDO₂i: temperature-adjusted DO₂i; BSA: body surface area

Temperature (°C)	Linear DO₂i (mL/minute/m²)	Q10 DO₂i (mL/minute/m²)	taDO₂i/BSA (mL/minute/m²)	taDO₂i total (mL/minute)
25	84	162.5	110.2	198.4
28	138	189.4	151.6	272.8
30	174	209.8	179.7	323.4
33	228	244.6	222.7	400.9
35	264	270.9	252.0	453.5
37	300	300	281.7	507.0

Statistical validation

The quadratic regression used to develop the taDO₂i model was derived from a simulated dataset informed by physiological data across temperatures from 24°C to 37°C, assuming a constant BSA of 1.8 m². The regression analysis yielded an R² value of 0.996, indicating excellent model fit. The standard error of estimate was 3.2 mL/minute/m². All coefficients (a = 0.057, b = 10.754, c = -194.245) were statistically significant with p values <0.001. Confidence intervals for the coefficients were calculated using 1,000 bootstrap resamples and are available upon request. To visually express the model’s reliability and variability, future graphical outputs will include error bars derived from the interquartile ranges of the modeled data. These additions will enhance the interpretability of the graphs by reflecting physiologic dispersion around predicted values at each temperature point.

Interpretation and algorithmic implications

**Figure 2:**
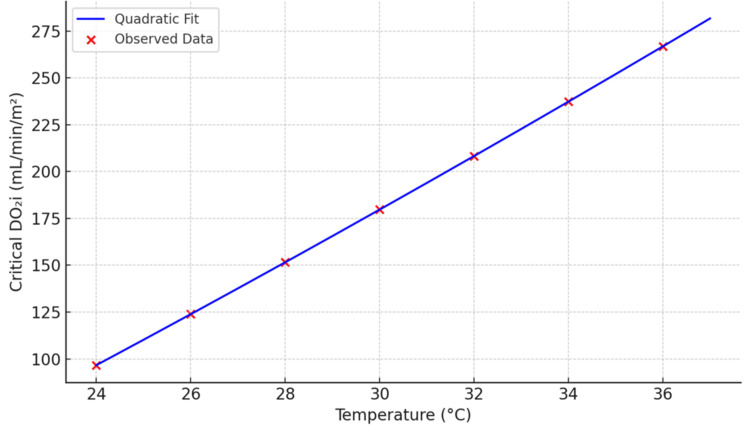
Quadratic regression model of critical DO₂i as a function of core temperature Red crosses represent observed or estimated critical DO₂i values at various temperatures. The blue curve indicates the best fit quadratic regression, described by the equation: DO_₂i_ = 0.057x² + 10.754x − 194.245, where x is the core temperature in degrees Celsius, and DO₂i is expressed in mL/minute/m². This model demonstrates a strong temperature-dependent relationship, with increasing oxygen delivery requirements as temperature approaches normothermia DO₂i: indexed oxygen delivery

The taDO₂i model effectively bridges the gap between empirical data and theoretical physiology. Unlike fixed thresholds, it dynamically modulates oxygen delivery targets based on the metabolic rate changes driven by temperature, with adjustments for patient size. This tailored approach is particularly valuable during CPB phases such as cooling, where unnecessary overperfusion may be avoided; hypothermia maintenance, where perfusion can be safely reduced; and rewarming, where rapid increases in VO₂ demand precise escalation in DO₂i. The quadratic behavior of the taDO₂i curve ensures a smooth, nonlinear transition between these states, minimizing the risk of perfusion mismatch (Figure 2).

## Discussion

Importance of DO₂i during CPB

Adequate DO₂i during CPB is essential to ensure metabolic homeostasis and prevent complications such as tissue hypoxia, organ dysfunction, and postoperative morbidity. Traditional GDP protocols have relied on fixed critical DO₂i thresholds, typically in the range of 272-300 mL/minute/m², based on retrospective associations with lactic acidosis and renal injury under normothermic conditions [1,2].

Limitations of static targets

These static targets, however, do not account for dynamic changes in metabolic demand that occur across the thermal phases of CPB [12]. The strong influence of core temperature on oxygen consumption (VO₂) has been acknowledged in various physiological studies, yet current perfusion strategies continue to apply fixed thresholds across the entire temperature spectrum [13].

Clinical risks across temperature ranges

This leads to two clinically relevant concerns: the risk of overperfusion during hypothermia, contributing to hemodilution and increased shear stress, and underperfusion during rewarming, with potential for tissue ischemia and organ dysfunction [14].

Development of the taDO₂i model

To address this gap, we developed a physiologically informed taDO₂i model based on a quadratic function. This model modifies critical DO₂i values in real time, using core temperature and BSA as inputs. It aligns with established metabolic principles such as the Q10 effect and Van’t Hoff’s rule but offers superior precision by integrating empirical fitting through regression analysis.

Model results and temperature-specific DO₂i

The model demonstrated that at core temperatures of 28-30°C, oxygen delivery requirements could be safely reduced to approximately 150-200 mL/minute/m², whereas at 36-37°C, values should return to normothermic standards (300 mL/minute/m²) [15]. A key innovation of this work is the algorithmic formulation of DO₂i targets, which permits continuous, individualized adaptation of perfusion goals during all CPB phases [16].

Real-time integration and precision perfusion

Unlike previous models, which were descriptive or theoretical, the taDO₂i algorithm is constructed for real-time clinical integration into heart-lung machine interfaces and electronic perfusion records. This represents a significant advancement toward precision perfusion [17].

Gaps in the current literature

To date, the literature lacks a robust, validated computational framework specifically designed to dynamically adjust DO₂i targets during CPB according to temperature-dependent metabolic demand. While the effects of hypothermia on VO₂ have been explored, no previous study has translated these physiological principles into a functional, bedside-deployable algorithm with the intent of optimizing perfusion practice [18,19].

Limitations and future directions

While the proposed taDO₂i model is grounded in well-established physiological theory and empirical fitting, its clinical applicability remains theoretical at this stage. Prospective clinical trials are required to validate the model’s safety, efficacy, and association with clinical outcomes such as lactate clearance, renal function, and postoperative recovery. Furthermore, integration into clinical workflow will necessitate compatibility with existing monitoring systems and perfusion software. In addition, the current model assumes a fixed relationship between temperature and VO₂ across all patient populations, which may not fully capture interindividual variability due to comorbidities, pharmacologic interventions, or inflammatory states. Future iterations of the model may incorporate real-time VO₂ or VCO₂ monitoring to individualize perfusion targets further.

## Conclusions

In this article, we have proposed a taDO₂i model that offers a physiologically accurate and dynamically adaptive framework for guiding perfusion during CPB. By incorporating core temperature and BSA into its predictive structure, the model provides superior alignment between oxygen delivery and metabolic demand compared to fixed-threshold strategies. Its computational simplicity facilitates potential integration into contemporary perfusion platforms and electronic clinical support systems. However, further clinical validation through prospective studies is necessary to confirm its efficacy and evaluate its impact on metabolic parameters, organ function, and postoperative outcomes. This work represents a foundational step toward individualized, temperature-responsive perfusion in cardiac surgery.
